# Intussusception in the ascending colon caused by cecal chronic diverticulitis: a case report

**DOI:** 10.1093/jscr/rjac204

**Published:** 2022-05-31

**Authors:** Tabito Oyama, Tomonori Akagi, Tsuyoshi Etoh, Hidefumi Shiroshita, Shota Amano, Hajime Fujishima, Yoko Kawano, Yohei Kono, Kosuke Suzuki, Shigeo Ninomiya, Tomotaka Shibata, Yoshitake Ueda, Norio Shiraishi, Masafumi Inomata

**Affiliations:** 1 Department of Gastroenterological and Pediatric Surgery, Oita University Faculty of Medicine, Japan; 2 Department of Comprehensive Surgery for Community Medicine, Faculty of Medicine, Oita University, Oita, Japan

## Abstract

Although intussusception in adults generally results from malignant tumors and polyps, intussusception caused by chronic diverticulitis is extremely rare. We present the case of a 59-year-old man treated with steroids and biopharmaceuticals at the Department of Dermatology for psoriasis vulgaris. Cecal diverticulitis frequently recurred, for which he was treated during follow-up. This time, endoscopy showed a circumferential stenosis in the ascending colon, and computed tomography showed the appearance of a target sign in the right colon. As a result, the patient was preoperatively diagnosed as having intussusception of the ascending colon triggered by diverticulitis of the cecum and underwent laparoscopic right colectomy. Pathological findings of the specimen revealed multiple diverticulitis of the cecum. Therefore, we thought that the intussusception was caused by chronic inflammation of the diverticula and surrounding fibrosis. Among intussusception in adults, chronic diverticulitis should be considered as a target of surgical treatment on rare occasions.

## INTRODUCTION

Colonic intussusception caused by chronic diverticulitis except for Meckel’s diverticulum is extremely rare. Intussusception often develops in early childhood in about 90% of cases, but adult cases are relatively rare at 6% of all cases of intussusception [1]. Most adult cases have malignant tumors (42–62%), followed by benign tumors (30–38%), idiopathic disease (8–17%) and Meckel’s diverticulum (3%) [2], and intussusception with diverticulitis is extremely rare. To treat intussusception, endoscopic reduction is first considered, followed by surgical colectomy if the endoscopic approach fails.

Although several rare cases of intussusception due to Meckel’s diverticulum, small intestinal diverticulum and appendiceal diverticulum were reported, few reports exist on intussusception of the colon caused by diverticula except for Meckel’s diverticulum. To our best knowledge, there are only three case reports of patients from Japan diagnosed as having intussusception of the colon caused by diverticula other than Meckel’s diverticulum [1, 8, 9], all in Japanese. The patient reported by Kudo *et al*. [1] underwent laparoscopic surgery; the other two were treated without surgery.

We report a rare case of intussusception into the ascending colon that developed in the cecum due to cecal diverticulitis and was successfully treated by laparoscopic surgery.

## CASE PRESENTATION

**Figure 1:**
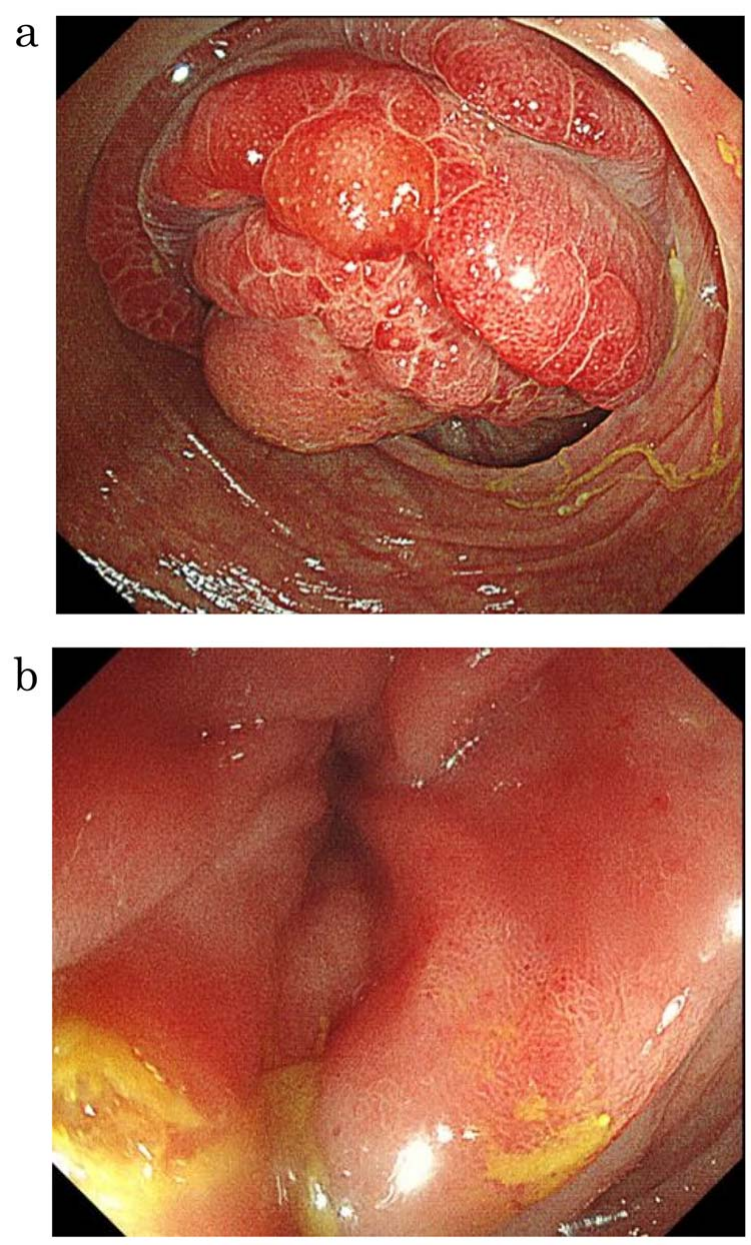
Lower gastrointestinal endoscopy showed a mass with circumferential stenosis in the ascending colon (**a**), and its surface showed vascular congestion without malignant findings (**b**).

**Figure 2:**
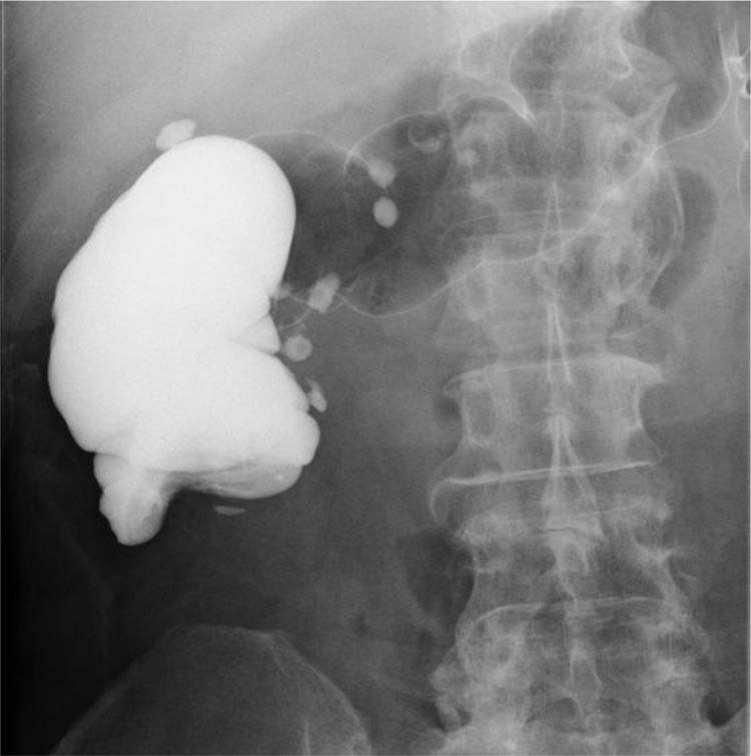
Enema colonography showed complete stenosis.

**Figure 3:**
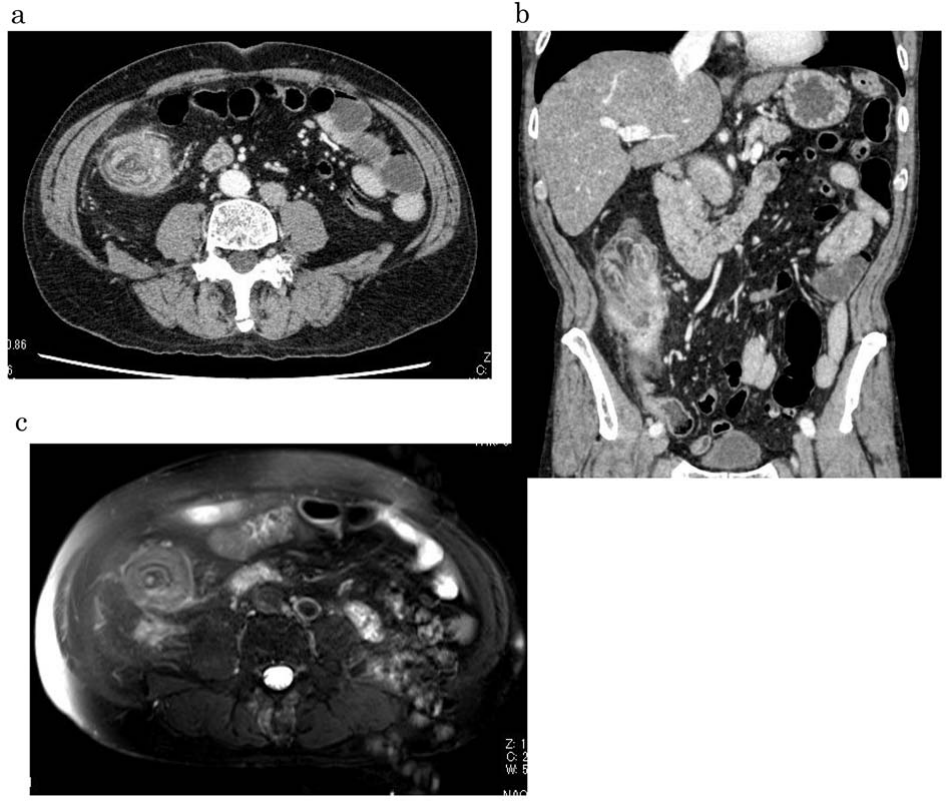
Contrast-enhanced CT/MRI showed increased wall thickness from the ileocecal region to the ascending colon (**a, b**), which was accompanied by a ring-shaped and layered appearance of the mucosal and muscular layers (**c**).

A 59-year-old man was followed up for a year by a dermatologist for psoriasis vulgaris. During this time, he suffered repeated occurrences of cecal diverticulitis diagnosed by contrast-enhanced computed tomography (CT) and was referred to our department for detailed examination and treatment. On examination, he had symptoms associated with subileus such as abdominal distension and pain. Colonoscopy showed a mass with circumferential stenosis in the ascending colon. The surface of the mass showed vascular congestion without malignant findings (Fig. 1a,b). An endoscope could not pass through the stenosis, and enema colonography showed complete stenosis (Fig. 2). Contrast-enhanced CT/magnetic resonance imaging (MRI) revealed a thick wall from the ileocecal region to the ascending colon, which was accompanied by a ring-shaped, layered appearance of mucosal and muscular layers (Fig. 3a–c).

**Figure 4:**
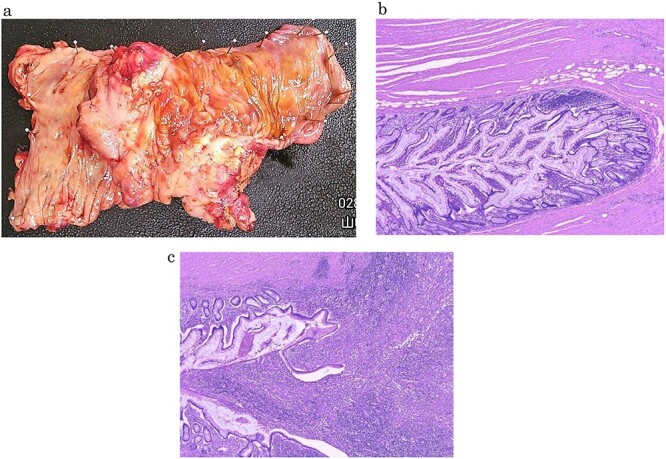
Resected specimen and photomicrographs of the pathology (**a**) The cecum and ascending colon showed remarkable diverticulitis that was accompanied by prominent wall thickening; the cecum was considered to be the front component of intussusception, and the internal lumen was stenotic due to organization **(b, c**) Pathological examination revealed the presence of abscess formation due to chronic diverticulitis and penetration by the diverticulum.

The patient was diagnosed as having ascending colonic intussusception due to chronic diverticulitis of the cecum. As the intussusception was not treatable by an endoscopic procedure, we performed laparoscopic right colectomy. The intraoperative findings showed that the wall of the ascending colon was thick, similar to a mass. However, the intussusception had been already released. As we thought it unlikely for the patient to have a malignant tumor based on the preoperative examination and intraoperative findings, we decided not to dissect the lymph nodes and performed right colectomy laparoscopically. After retrieval and examination of the specimen in detail, we found that the diverticulum of the cecum communicated with the terminal ileum to form a fistula. In addition, the front part of the intussusception in the ascending colon was the cecum. An abscess surrounding the ascending colon showing intussusception was also present (Fig. 4a).

The resected specimen contained several diverticula from the cecum to ascending colon, with a relatively hard mass of 1.5 cm in diameter on the serosal side of the cecum. The cecum and ascending colon showed remarkable diverticulitis accompanied by prominent wall thickening. The cecum was considered the front component of intussusception, and the internal lumen was stenotic due to organization (Fig. 4a). Pathological examination revealed abscess formation due to chronic diverticulitis and penetration by the diverticulum (Fig. 4b,c). Follicular hyperplasia was observed but without atypia, and histology was consistent with reactive hyperplasia associated with inflammation. The specimen showed no malignant findings.

The patient was discharged from hospital 7 days postoperatively without any complications.

## DISCUSSION

Colonic intussusception caused by chronic diverticulitis except for Meckel’s diverticulum is extremely rare. To our knowledge, this is the first adult case of cecal intussusception caused by cecal diverticulitis reported in English to be successfully treated by laparoscopic surgery.

Intussusception is classified as small intestine type in 36.7%, ileocecal type (ileal colon type, cecal colon type) in 44.7% and colon type in 19.1% of cases. Most pediatric cases are idiopathic, but most adult cases have malignant tumors (42–62%), followed by benign tumors (30–38%), idiopathic disease (8–17%) and Meckel’s diverticulum (3%) [2]. In a 16-year retrospective study of adult intussusception, Honjo *et al*. [3] found that intussusception was due to neoplastic lesions in 77.3% of 44 patients and malignant tumors in 73.5%, with intussusception due to diverticula being extremely rare except for that caused by Meckel’s diverticulum.

Colon diverticulum increases with age, and it prevalence is increasing in Japan. In a study of 28 192 patients who underwent colonoscopy from 2003 to 2011 [4], 21.2% had a colonic diverticulum, with 16–22% being under the age of 40 and 42–60% being over the age of 80 years [5]. About 80 to 85% of patients with colonic diverticulum spend their lives without symptoms. However, according to reports in Europe and the USA, diverticulitis occurs in 10–30% of patients [6, 7]. So far, excepting Meckel’s diverticulum, only one patient has been reported to have undergone surgery for intussusception caused by a diverticulum [8]. Generally, reduction by barium enema examination or endoscopy is performed first. If reduction is difficult or intussusception recurs, surgery is indicated. The present case was thought to have been caused by a mass due to abscess formation from diverticulitis that led to intussusception. The site of diverticulitis was in the cecum, which was relatively mobile, and was also thought to be a cause of intussusception.

Our patient’s endoscopic macroscopic findings showed vascular distension and redness at the mucosal surface and circular stenosis. CT and MRI images showed ring-shaped and layered mucosal and muscular layers, suggesting a target sign. Because reduction by barium enema examination and endoscopy could not be achieved, we performed intestinal resection of the stenotic site including the diverticulitis via a surgical procedure. During the operation, we were alert to the potential for adhesions between the intestinal walls and surrounding tissues related to the inflammation and abscess of the diverticulitis, which caused reduction through nonsurgical conservative treatment to be difficult. The present patient’s pathophysiology should be kept in mind as one differential diagnosis of intestinal wall thickening in patients with chronic diverticulitis.

## CONCLUSION

We treated a patient with chronic diverticulitis with intussusception. In adults with intussusception, chronic diverticulitis should be considered as a target of surgical treatment on rare occasions.
